# Telework: A Social and Emotional Perspective of the Impact on Employees’ Wellbeing in the COVID-19 Pandemic

**DOI:** 10.3390/ijerph20031811

**Published:** 2023-01-18

**Authors:** Monica Aureliana Petcu, Maria Iulia Sobolevschi-David, Raluca Florentina Crețu, Stefania Cristina Curea, Anca Maria Hristea, Mihaela Diana Oancea-Negescu, Daniela Tutui

**Affiliations:** Department of Financial and Economic Analysis and Valuation, Bucharest University of Economic Studies, 010374 Bucharest, Romania

**Keywords:** telework, wellbeing, COVID-19 pandemic, autonomy, emotional dimension, organization, work intensity, relational communication, work–life balance

## Abstract

The COVID-19 pandemic gives us the largest telework experiment ever conducted globally, that will most likely leave visible and lasting marks on the organization of the labor market in the future. The purpose of this approach is to investigate the wellbeing from the social and emotional perspective of the individual, considering relevant relational communication, emotional dimension, work intensity, organization, autonomy and work–life balance, customized in the context of teleworking. The information was collected using a semi-structured questionnaire. The wellbeing assessment was performed based on the correlation analysis and the regression analysis. The results of the studies reveal that the existence of adequate communication and work–life balance ensure the wellbeing of telework employees, while the increase in work intensity degrades it. Furthermore, good communication moderates the relationship between organizational skills and wellbeing. The comparative analysis of wellbeing in relation to the explanatory variables considered by including the gender and age perspective reveals the existence of different configurations, with specific signs and statistical meanings.

## 1. Introduction

In the last three decades, telework has become a challenging topic, proven both by its academic relevance attested by numerous papers, studies and new approaches presented in the literature, and especially by the practical implications it generates on the strategic management of companies.

Since the spring of 2020, the rapid spread of the new SARS-CoV-2 virus has required government officials in many states to find quick solutions for companies to implement measures to keep employees at bay to prevent disease, where the field of activity is enabled.

Remote work is a reality of the present with advantages and disadvantages, both for the employers and for the employees. The positive effects of teleworking on employees include increasing productivity, reducing absenteeism, improving digital skills, increasing the possibility of maintaining employment, reducing time loss caused by commuting, enhancing flexibility of the work schedule, and allowing the possibility of working from any place. Among the negative effects, the most frequently mentioned by teleworkers are impairment of the psychological component (anxiety, exhaustion, depression, social disconnection, mental fatigue, dissatisfaction, loneliness, sedentary lifestyle), imbalance between personal and professional life (extended work schedule without additional incentives, interruptions in work/concentration rhythms due to interaction with some family members), increased personal costs (with electricity, internet, purchase of devices), increased responsibilities of women in family life, increased work dependence, reduced free time spent with family, reduced interaction with friends, etc. The literature suggests that no perfect hologram will ever be able to replace face-to-face interaction. For e-workers, professional activity will increasingly overlap with home life and working time and lack of free time can become a reason for bargaining between employers and employees [1].

Employee wellbeing is approached separately by economists, sociologists and psychologists. Thus, economists perceive it in terms of salaries, working hours, the possibility of promotion, social and health insurance. Sociologists include in the evaluation aspects related to control and autonomy, as well as personal development. From a psychological point of view, the relationships within the entity, and the feeling of trust and security conferred by it, are considered [2].

The aim of this approach is to analyze the social and emotional impact of telework on employees as determinants: work intensity, relational communication, emotional dimension, organization, autonomy and work–life balance, customized in the context of the COVID-19 pandemic.

Considered are the following research questions: RQ1: Does a high work intensity affect the wellbeing of telework employees?; RQ2: Does clear and regular communication guarantee an increased wellbeing of telecommuting employees?; RQ3: Is the emotional dimension a predictor of wellbeing?; RQ4: Does the ability to efficiently organize the activity ensure the wellbeing of employees?; RQ5: Does a high degree of autonomy constitute the premise of a higher wellbeing of employees in remote work?; RQ6: Is the work–life balance a condition for a higher wellbeing of telework employees?

This research theoretically contributes to the development of a conceptual approach and the measurement of employee wellbeing, with particularization in the case of telework, by describing the explanatory factors and including them in an integrative model, which validates the partial results obtained by other researchers. Furthermore, the proposed multidimensional model allows the substantiation of the measures on labor design at the level of employers and reconsideration of policy objectives at the macroeconomic level, by including the quality of working life in order to improve wellbeing as a condition of sustainable development. In addition, the deepening of the analysis from the perspective of gender and age offers benchmarks to managers, allowing them to take measures to capitalize individual potentialities and reduce adverse effects generated by telework. The study is a continuation of the research conducted in 2021 by some of the authors of this paper.

This paper is organized as follows. In the next section, a review of the literature in the field is made, specifying the concerns regarding the conceptualization and wellbeing measurements. Section 3 presents the variables of the wellbeing assessment model and how they create wellbeing and job satisfaction as you move from the traditional office setting to an alternative work environment. The following sections focus on the research methodology, the results of the empirical research and the discussions on them; then, the authors finally present the main conclusions of the study.

## 2. Literature Review

Work wellbeing is a construct that provokes a continuing interest in research, especially since there is no clear and convergent definition [3,4]. Research emphasizes that the notion of work wellbeing has its roots in the theory of wellbeing [5,6] developed in the field of psychology. Welfare as a balance between personal psychological, social and physical resources and the challenges of the same nature that a person faces [7] is a definition that affirms the complexity of the concept.

The conceptualization and measurement of the wellbeing construct continuously involves a variety of determinants, identifying the high interest for its study. Lambert et al. [8] examined the proposals of the August 2019 Kyoto International Conference on the introduction of new wellbeing variables, considering that wellbeing reflects, in addition to life satisfaction, the hedonic and eudaimonic component, social welfare, and cultural aspects related to the community, nature and governance. In the context in which people allocate a large part of their time to work, the inclusion of the concepts proposed by the Greek philosophers, respectively, hedonism, as a reference to maximizing pleasure, and eudaimonia, as the perspective of achieving happiness in accordance with their own virtutes, are relevant in ensuring employees’ wellbeing. Studies show a diverse modeling of the construct over the last decades [5,9,10,11] and in a continuous dynamic, the perspectives approached in the psychological field closely support workplace wellbeing assessments from research over time [4,5,12,13]. A holistic approach to work wellbeing [3] is argued by the consideration regarding the strategic nature of the construct in the decision-making processes. It has been found that most contemporary work wellbeing definitions use subjective emotion as a general indicator. Thus, work wellbeing can be connected both with the concept of subjective wellbeing, which refers to people’s cognitive and affective assessments of their lives, and with affective wellbeing, related to the different experiences that an individual has, which are determined by emotions, in these connections being present in the concept of psychological wellbeing [14]. These components of work wellbeing are interconnected and fit the concept into wellness theories, rather than the traditional theory of stress.

Numerous studies consider wellbeing at work to be an objective of human resource management, being a predictor for employee performance, and others relate the quality of professional life to the quality of personal life. Research shows a correlation between subjective wellbeing and job performance, although the causality of that relationship is considered questionable [15]. Other researchers [12] have built a model linking workforce wellbeing, productivity and population wellbeing. National welfare policies should take into account the quality of working conditions and the factors that favor positive personal relationships [16], as the psychosocial work environment and personal relationships are determinants of subjective wellbeing.

Understanding the interrelationships between workplace wellbeing and subjective wellbeing is essential to designing occupational health and safety policies, but also to improving employee satisfaction and wellbeing [17]. Occupational safety and health are considered to be determinants of employee performance. Pagán-Castaño et al. [18] studied employee wellbeing as a moderating variable between human resource management and organizational performance.

Highlighting the impact of wellbeing at work on organizational performance has led many researchers to identify the determinants of the construct. Page and Vella-Brodrick [13] proposed an integrative model, in which employee wellbeing has three components, namely subjective wellbeing, workplace wellbeing and psychological wellbeing, and influences organizational wellbeing, perceived through staff fluctuations and their performance. Happiness at work includes employee satisfaction, but it is much more than that [4] and includes commitment to work or company. The inclusion of these variables in the model of wellbeing at work has attracted the attention of researchers to the eudaimonic perspective [6], developing a two-dimensional scale of wellbeing at work (Eudaimonic Workplace Wellbeing Scale (EWWS): interpersonal workplace wellbeing and intrapersonal workplace wellbeing.

The Job Demands-Resources (JD-R) model, advanced by Bakker et al. [19], has been used in the field of occupational health and safety worldwide, with the welfare of work being conceptualized as a function of the compromise between demands and resources. The resources (autonomy, variety of skills, feedback on performance and growth opportunities) involved in work, as well as work-related demands, are physical, psychological, social or organizational components that are related to achieving work goals, reducing physiological and psychological effort and costs associated, and stimulation of personal growth and development [20]. Research conducted over time by the authors of the model has shown that excessive work-related tasks, emotional demands on employees, physical demands on the job, and impairment of employees’ personal relationships with family are all risk factors for employee exhaustion. Their unwanted effect can be mitigated by work resources such as job autonomy, social support, quality of the relationship with the supervisor and performance feedback.

Studies conducted prior to the forced proliferation of telework in 2020 have shown specific determinants of the wellbeing of telework employees. Social support from colleagues, participation in decision-making, autonomy in carrying out tasks and work in a family context (work-to-family conflict), aspects that define work characteristics, were directly related to work-related wellbeing, and the teleworking has been indirectly linked to welfare through social support [21,22].

Studies on employee wellbeing, in the context of forced adoption of telework, have focused on variables related to mental health. One of the first studies to show the consequences of isolating employees, in the context of the COVID-19 pandemic, on employee wellbeing associated employees’ psychological stress (poor wellbeing) with education, habits, and higher work intensity during the pandemic, lifestyle and psychological distress [23]. An analysis of the effects of remote work on the psychological health (psychosomatic manifestations) of employees by applying the statistical method of path analysis on a large sample of data [24] identified indirect relationships between remote work and employee health, moderated through variables such as working time control, time pressure, boundaryless working hours, relationships with co-workers and disturbances and interruptions. High control of the workplace has been positively linked to emotional exhaustion and work–life balance [25].

In the literature, perceived wellbeing in the workplace can be linked to many factors [26], such as training, general work environment, level of gender equality. Greater wellbeing at work [27] is reflected in higher overall wellbeing of employees. It was found that women’s perceptions of all variables studied are lower than those of men; there is a causal relationship between perceived gender equality, training and wellbeing at work, but especially between the latter variable and general wellbeing. Work–life balance [28] is a major issue for organizations and employees. They benefit from better health and wellbeing when they have achieved this balance. Control variables that influence work–life balance include age, working time, level of education and household income [29]. Although these variables have a moderate effect on the health and wellbeing of employees, they have a direct impact on employee productivity and the results of organizations.

A study conducted in 2017 by Williams et al. [30] draws attention to the importance of developing, on the one hand, the positive attitudes of employees, and on the other hand, the organizational culture [31] on developing employee happiness at work. The literature improves from year to year with studies on employee wellbeing and human resource management by integrating intrinsic motivation as a model of mediation in labor relations [32]. The effects of the interaction between different socially responsible human resource management practices on three variables of employee wellbeing: job satisfaction, stress at work, and trust in management are topics to be analyzed in periods of recession and economic crises [33]. An important role during or outside economic crises is played by time management, which seems to improve wellbeing—especially life satisfaction—to a greater extent than performance does. This raises the perception of many researchers [34] that time management primarily improves performance in the workplace and that wellbeing at work is simply a by-product.

In Romania, Law no. 81/2018 regarding the regulation of telework activity, with subsequent amendments and additions, defines the terms and the ways of performing the activity. Telework is defined as the form of work organization through which the employee, on a regular and voluntary basis, fulfills the duties in a place other than the workplace organized by the employer, using information and communication technology. It is essential that this way of performing the activity should not be accidental, fortuitous, but that the employee should constantly perform at least part of the activity remotely. The performance of the activity is based on the voluntary agreement of the parties, which jointly establish the work schedule. Under the conditions established by the individual employment contract, the internal regulation and/or the applicable collective labor agreement, the employer has the right to verify the teleworker’s activity. Among the employer’s obligations, relevant in the context of the research, we mention the sufficient and adequate training of employees and importance taking measures so that the teleworker is not isolated from the rest of the employees, ensuring the opportunity to meet with colleagues regularly. The legal framework regarding teleworking was adapted to the conditions of the COVID-19 pandemic. Thus, according to DECREE no. 195 of 16 March 2020 regarding the establishment of the state of emergency on the territory of Romania, where possible, during the state of emergency, work at home or telework can be imposed by a unilateral act of the employer. LAW no. 55 of 15 May 2020, regarding some measures to prevent and combat the effects of the COVID-19 pandemic, provides that during the state of alert, employers can arrange work at home or telework, where the specifics of the activity allow, with the consent of the employee, in compliance with the provisions of Law no. 53/2003—Labor Code, republished, with subsequent amendments and additions, and of Law no. 81/2018 on the regulation of telework activity.

## 3. Determinants of Wellbeing from a Social and Emotional Perspective

The evaluation of wellbeing in the present research is made from the social and emotional perspective of the individual, considering relevant relational communication, emotional dimension, work intensity, organization, autonomy and work–life balance, customized in the context of teleworking during the COVID-19 pandemic.

Numerous research articles have highlighted the diversity of relational communication and its impact on the wellbeing of employees in the remote work system. Communication is the basis for creating and maintaining social relationships between employees, as well as between them and the organization. As teleworking has become a common organizational concept, many experts have looked at how communication and relationship variables create wellbeing and job satisfaction as we move from the traditional office setting to an alternative environment—distance work [35,36,37,38]. When working from home [39], employees may feel disconnected from their organizations; for a successful transition to telecommuting, effective organizational communication is essential. Despite the diversity of approaches, it was concluded that for employees with no previous work experience, relational communication in the context of COVID-19 [40], although significantly influenced by the organization’s culture, divides the portfolio into three categories: a group of employees who consider teleworking a difficult challenge for the development of inter-social relations and communication; a group that considers remote work an opportunity to increase wellbeing through communication and relationships; and an ambivalent group.

The issue of low support, unfavorable social climate, lack of procedure, existing labor disputes with superiors and colleagues are significant risk factors at work, making it necessary to study them [41].

Becoming a reality a few decades ago, telework has been analyzed over time from many perspectives [42], including global efficiency, organizational advantages and disadvantages, the attractiveness of employees to this kind of work, and productivity and performance. Although little attention has been paid to it in the past, the perception of the impact of telework on the emotional dimension has changed in recent years. Thus, before the onset of the pandemic, employees experienced more work-related emotional wellbeing in the days when they worked in the telework system, compared to the days when they worked in the office [42]. The COVID-19 context has generated many fears and changes in the psychological wellbeing of employees with mass social isolation [43]; after the imposition of pandemic restrictions, teleworkers have experienced a lower degree of self-perceived emotional wellbeing in the last two years compared to employees who continued their work in the traditional office system [44,45]. Most of the time, the emotional dimension is more strongly felt by women, whose family obligations have increased significantly with the transition to the online work system. Furthermore, it differs depending on the nature of the job, the position held within the organization and the complexity of the tasks [46].

“Conceptually, work intensity is ambiguous, and has included elements, such as arising pressure of pace, time pressure, work overload, tight deadlines, harder work, and long and unsocial hours” [47]. It is necessary to distinguish between the pressure exerted on the work intensity of the skills held, and that related to additional efforts in the unit of time considered normal. From the perspective of this analysis, it is considered input to work tasks during the working day. As labor markets transition to more flexible working models based on digitization, labor intensity increases so that an increasing number of tasks can be performed and monitored anywhere and anytime with the help of new mobile information and communication technologies [46]. Relevant studies show that compared to office work, teleworkers face an increase in work intensity, frequent interruptions during work, extended working hours, reduced or even lack of time for recreation, and more requests to work in spare time [48], with high speed and short deadlines for tasks. All this induces stress and diminishes the wellbeing of teleworkers [44]. The pandemic rightly created a surreal work environment [49], characterized on the one hand by constraints to maintain social distancing, and on the other hand by pressures to maintain the connection between employees and the organization, which led to work intensity increases most of the time.

Anticipating radical changes in the way work was organized decades ago, Alvin Toffler pointed out that with the evolution of technology, work would no longer necessarily take place in offices or factories. “It will take place anywhere, anytime” [50]. Remote work requires more organization that can increase productivity, especially in independent tasks that require minimal coordination with colleagues [51], but the social isolation generated by teleworking is often cited as a negative aspect of wellbeing [52].

Productivity and wellbeing are far from reflecting the sufficiency and self-satisfaction of remote work [53], autonomy being considered a key complementary variable. The degree to which an individual can exercise choice and initiative at work is referred to as autonomy, being determined by how work is organized, particularly the extent to which work processes are standardized and whether work tasks are carried out under the rules and procedures or surveillance systems [47]. In a general sense, autonomy refers to the degree of flexibility that the employee has in planning and organizing work, as well as the means one can use to achieve their own goals. Autonomy means being at the helm of own experiences and actions, having a high degree of independence and responsibility in projects [54]. In approaches to the theory of intrinsic motivation and self-determination in human behavior, studies of the brain have shown that “people who feel more autonomous make better decisions” [55]. Numerous research carried out in the last decades highlight the fact that there is a correlation between autonomy at work and wellbeing. Autonomy is the key engine of human motivation, performance and fulfillment [56], which is closely correlated with happiness, responsibility, creativity, skill development and productivity. In the context of hybrid work and telework, the employee is strongly interested in being, themselves, the main decision maker in choosing the place and time of work.

There are many theories interpreting the relationships between work and family life [46]. Among them, role theory, (inter)role conflict theory and boundary/border theory provide the basic terminology and perspectives to understand other theories [46,57,58]. One of the main explanations for why working remotely does not help reduce the conflict between work and life is related to the loss of the boundaries between work and family, manifested by a significant increase in family responsibility that can amplify the conflict between these two roles [59]. Work–life balance should be considered a priority [60]. Many employees believe that through telework, professional and family roles often come into conflict [61], and the work–life dimension is rather a variable with a negative impact on wellbeing. The concept of work–family conflict is becoming more common lately because “mutually incompatible pressures on the simultaneous position of telemployee and family member cause conflicts and make it difficult to meet expectations in either family or professional life” [62].

Emotions manifest in different ways, depending on cultural and contextual factors, targeted by motivational determinants in adopting a specific behavior [63]. Emotional wellbeing consists of the ability to generate positive thoughts and to adapt when faced with adversity and stress. At work, emotions will materialize in a greater or lesser commitment to the employer.

## 4. Research Methodology

In order to analyze the impact of work intensity, relational communication, emotion-al dimension, organization, autonomy and work–life balance on wellbeing, data were collected through a social survey based on a semi-structured questionnaire, in which the first 5 items are intended to present the respondents (field of activity, age, gender, education and training, work regime), and the following 24 items aim to characterize the personal state through the explanatory variables. For this last category of items, the default answers were very much, much, enough, little, not at all, which were converted to a Likert scale from 1 to 5. The questionnaire was distributed randomly in Romania, in the COVID-19 pandemic period, selecting only the answers of the employees who carry out their activity in the telework regime. 

In the sampling design process, we considered the active population in Romania, which, according to the National Institute of Statistics, decreased from 8736.9 thousand in 2020 to 7835.6 thousand in 2021. During the COVID-19 pandemic, the number of the population working in a telework regime (the target population) varied between a minimum of 62,209 and a maximum of 435,367 according to the National Institute of Statistics, which corresponds to a weight of 5.6%. The sample size is determined using Cochran’s Formula for Sample Size, considered appropriate for large populations that are heterogeneous in nature, at a 95% confidence level, (which gives a Z values of 1.96, per the normal tables), with +/−5% margin of error. The analyzed sample is 440, consisting of respondents working in various fields of activity (banking system, education, consulting, HR, accounting, audit, research, public administration, trade, IT, tourism, etc.), with a share of 33.18% of men and 66.82% of women. Of the total number of respondents, 52 respondents were up to 25 years old, 116 were between 26 and 35 years old, 192 were between 36 and 50, and 80 were over 50 years old. We consider the size of the sample studied to be relevant, including a number greater than that determined by applying Cochran’s Formula for Sample Size.

To evaluate the data used, in the exploration and formulation of the conclusions, we used descriptive statistics (mean, median, maximum, minimum, Std. Dev.). These indicators, calculated on the total and on the considered subsamples, allow the characterization of the respondents’ perception regarding the variables that we consider of interest and the differences between them.

The measurement of the strength and direction of the linear association between variables is performed with the help of the Spearman coefficient, with no assumption of causality.

Internal consistency analysis was performed using Cronbach’s alpha, which highlights how closely related a set of items are as a group. The Cronbach’s alpha coefficient is 0.83, higher than 0.70, considered acceptable in social science research, revealing a high internal consistency of the variables.

The assessment of the wellbeing was made on the basis of the correlation analysis and the regression analysis, using the EViews program. The variables are presented in Table 1.

In order to evaluate the intensity of work, questions regarding the concern for work tasks outside the formal work schedule, the intensity of feeling deadlines, additional requests and the degree of exhaustion generated by permanent access to work were included in the questionnaire. Developing the ability to socialize online, easy communication with colleagues and the team manager/coordinator are elements of the workplace relationships and communication assessment. The evaluation of the emotional dimension is conducted based on the questions regarding the states of anxiety, fatigue, pleasure and satisfaction felt in the case of telework. The answers to the questions considering the ability to work individually and to effectively manage the work schedule allow an appreciation of the ability to organize the activity. The degree of decision freedom in the management of one’s own activity, the possibility of opting for a flexible program, the existence of procedures regarding the development of activities and the measure of exercising control are aspects that allow the evaluation of the degree of autonomy. The assessment of work–life balance is carried out on the basis of questions aimed at the degree of suitability of the flexibility of the work schedule to family life and other activities, the extent to which the disturbance from the activities, as a result of interruptions determined by solving family problems, affects the quality of work and the level of satisfaction in terms of work–non-work time balance. 

In view of the general appreciation of wellbeing in relation to the work performed, a distinct question was asked, which would allow the evaluation of the employees’ perception as a result of experimenting with telework. 

The next stage of the analysis is carried out with the regression method, from the literature review noting the use of both the ordinary least squares (OLS) and the ordinal logit model (OLM).

In order to verify the assumption of the multiple linear model we provide some post estimation tests: heteroskedasticity (Breusch–Pagan–Godfrey test), non-correlation of errors (Lagrange Multiplier test), and non-collinearity of variables (VIF values of independent variables). 

Verifying the validity of the model by checking its robustness is achieved by applying the model to subsamples (at the gender level: women and men) and performing specific tests.

Also, the robustness is checked using OLM, specifically in the analysis of ordinal variables.

## 5. Empirical Findings and Discussions

The analysis of the data shows a favorable perception of wellbeing at the sample level, with higher values for men compared to those reported by women, for which the psychological component has a greater impact. This assessment remains relatively similar in all variables, with insignificant differences between women and men, with the mention that men are more satisfied with the organization and work–life balance. Only autonomy was not appreciated by any respondent, with the highest grade, but the overall average was slightly higher than that for work intensity, which was the lowest. The organization of the activity is appreciated most favorably by the employees in the sample. Although the standard deviation sample is small, there are differences between both women and men (Table 2).

The first objective of the study is to analyze the correlations between the variables. Table 3 points out the results.

At the level of the entire analyzed sample, it is observed that the existence of positive correlations is statistically significant between the variables of the model. The exception is the correlation between autonomy and work intensity, as well as between wellbeing and work intensity, which are statistically insignificant. Regarding the intensity of the relationship, the data reveal that high values of wellbeing are associated with higher values of the perception of the organization, as well as of communication and relationships at the level of the organization. There is a medium intensity correlation between communication and relationships, autonomy, as well as work–life balance and wellbeing at the level of the organization. Furthermore, in terms of emotional exhaustion, there is a high correlation between this and work intensity, as well as with work–life balance. There is a medium correlation between autonomy and relational communication, between autonomy and work organization, as well as between work intensity and work–life balance.

Study 1—Determinisms in relationship socio-emotional dimensions and wellbeing.

Estimates for the regression equation are presented in Table 4.

It is found that the sign and the statistical significance in the case of each independent variable are maintained in the two models used.

Work intensity has a negative, statistically significant effect on wellbeing (RQ1). This negative association is in accordance with the results obtained by Avgoustaki and Frankort [64], Meyer and Hünefeld [65], Felstead et al. [48], and Boxall and Macky [66]. Higher levels of work intensity degrade employee wellbeing. The constant access to work is exhausting; the flexibility of the program predisposes the employees in the telework regime to carry out their activity and to think about the work tasks outside the normal working hours. The appearance of additional requests and changes, as well as the deadlines for completing the tasks, are felt by the employees, affecting their wellbeing.

Emotional dimension influences wellbeing, observing a positive relationship, statistically significant (RQ3). The respondents do not report a state of anxiety, insecurity in the conditions of work, the work in this regime giving them a state of satisfaction and optimism, as well as the pleasure of having freedom over the work schedule. Our results are similar to those of Shama Kadadi et al. [67] and Anderson et al. [42].

There is a positive statistically significant relationship between business organization and wellbeing (RQ4). Respondents have the ability to work alone, to manage their work schedule efficiently and benefit from an organization of activity by the employer that supports efficiency and wellbeing.

Autonomy has a positive, statistically insignificant effect on wellbeing (RQ5). The telework experience involved a high level of autonomy, imposing a new design of work in terms of time and activity customized at the individual level, with benefits on employee performance and wellbeing. Our results are similar to those of Babapour Chafi et al. [68], Charalampous et al. [45], Curzi et al. [44], Meyer and Hünefeld [65], Boxall and Macky [66]. The employees benefit from a high level of freedom of decision in the management of their own activity and from the possibility of a flexible program. Employers do not exercise permanent control over the fulfillment of their duties and the deadlines for their performance, emphasizing the development of a system of procedures that govern the conduct of business.

The work–life balance ensures an improvement in the wellbeing of employees, with a statistically significant link between them (RQ6). Similar associations are found in Haar et al. [69], Arif and Farooqi [70], and Ter Hoeven and Van Zoonen [71]. On the one hand, the flexibility of the program ensures the adequacy of the work program to family life and other activities, and on the other hand, the respondents did not report dissatisfaction with the difficulty of performing work tasks due to family interruptions or family tasks, presenting a high level of work–non-work balance satisfaction.

Relational communication has a positive, statistically significant effect on wellbeing (RQ2). This is ensured by a development of the online socialization capacity as a result of the work regime, by the easy communication with the manager/coordinator of the team, as well as with colleagues. Giving the coordinator confidence in their own ability to perform tasks on time, as well as the necessary support for performance ensures the wellbeing of employees. Our results are similar to those obtained by Qin and Men [72], Jämsen et al. [40], Hager [73], and Collins et al. [74]. In this research we considered relevant only the communication and the relational support provided in the context of telework, the proactive work-support and good workplace-relationships, respectively, considering that telework allows employees to keep a distance from negative working relationships.

We performed the regression analysis considering that RC moderates the relationship between organization and wellbeing (the regression coefficient 0.074, *p* = 0.001). The issue of wellbeing in telework imposes additional capabilities for employees. If in the case of carrying out the activity at the company offices, the employees benefit from an adequate infrastructure, then in the case of telecommuting, the capitalization of the skills regarding the organization of the activity implies adequate communication with colleagues and superiors.

In order to verify the validity of the model, we checked its robustness by considering some subsamples (at the gender level: women and men, by age) and we performed specific tests. Highlighting the particular aspects of gender and age are important references for managers in promoting a participative management and developing the organizational culture that ensures the efficiency of the activity. Knowing the differences in the perception of women and men, as well as between people of different ages regarding the impact of telework, allows taking measures to capitalize individual potentialities and to reduce adverse effects.

Study 2—Determinisms in relationship socio-emotional dimensions and wellbeing by gender.

The inclusion of the gender perspective in the wellbeing evaluation highlights specific aspects, corresponding to the affective and social structures characteristics of women and men (Table 5).

It is found that the sign and the statistical significance in the case of each independent variable are maintained in the two models used.

The comparative analysis of female and male wellbeing points out differences in the meaning and significance of the impact of certain defining variables. There is a negative, statistically significant relationship between work intensity and wellbeing (RQ1). Men feel the pressure of deadlines more acutely. In addition, from this perspective, relational communication is considered essential for the wellbeing of respondents (RQ2). In contrast, the association between the emotional dimension and wellbeing is statistically significant only in the case of women, for whom teleworking confers the state of contentment and optimism (RQ3). The difference between the estimated coefficient for women and that for men in the case of organization is explained in the sample by the fact that men report a higher ability to work alone and a superior ability to effectively manage their program, with women’s efforts in this regard being felt stronger (RQ4). Regarding the effect of autonomy on wellbeing, the existence of procedures regarding the development of the activity is more important for men than for women. Furthermore, the impact of exercising permanent control over tasks and accomplishments is greater for men (RQ5). Work–life balance has a stronger effect, statistically significant, in the case of women, as a result of their multiple tasks in their double quality (RQ6).

Our results regarding the impact of telework on women and men are consistent with those from previous studies [75,76,77]. Approaching this issue at the level of the variables reveals the contribution of the paper in the field, the subject being studied to a lesser extent in the literature.

Study 3—Determinisms in relationship socio-emotional dimensions and wellbeing by age.

The assessment of wellbeing from the perspective of social and emotional variables by age categories highlights particular aspects (Table 6).

The comparative analysis of wellbeing in relation to the considered explanatory variables reveals the existence of different configurations, with specific statistical meanings and significances. The intensity of work is felt unfavorably by all people; the pressure of permanent access to work and the multitude of tasks affect wellbeing. Statistically significant correlations are registered only at the level of employees aged between 36 and 50 years (RQ1). The existence of good communication at the organizational level and the provision of support in fulfilling the tasks determine positive values of the coefficient of the relations within the organization. For employees over the age of 36, the confidence that the coordinator has in terms of their ability to perform their tasks according to the standards and at the deadlines generates higher values of coefficients and positive repercussions on wellbeing (RQ2). There is a positive relationship between the emotional dimension and wellbeing at the sample level, with the exception of the age group between 26 and 35, the insecurity and lack of satisfaction that telework confers constituting the main causes (RQ3). The reporting data show a positive link between organization and wellbeing, statistically significant in the case of employees aged between 26 and 50 years. The highest intensity is at the level of respondents aged between 26 and 35, who report a superior ability to work alone and to manage their work schedule efficiently (RQ4). Between autonomy and wellbeing there are particular situations by age groups. Employees under the age of 26 are satisfied with the possibility of benefiting from a flexible program, while for those aged between 26 and 35 there is a negative relationship, as a result of the pressure of control exercised over the accomplishment of tasks and deadlines. Statistically significant positive values are registered at the level of employees over the age of 35, for whom the competencies and experience give them the freedom to decide on the management of their own activity (RQ5). For employees up to the age of 25, the work–life balance has a negative effect on wellbeing, with them not being satisfied with the proportion of time allocated to work and non-work. For respondents between the ages of 26 and 50, work–life balance has a positive effect on wellbeing, which is statistically insignificant. Only at the level of the group of employees over the age of 50, the relationship becomes statistically significant, with the greatest impact on wellbeing. This is explained by the fact that this category of employees no longer feels the pressure of family tasks so strongly, not being disturbed from professional activity by the acuity of family tasks (RQ6).

The robustness of the model is tested using OLM (Table 7).

It is found that the sign and statistical significance, in the case of most independent variables, are maintained in the two models used. Differences are noticed regarding the sign of the work intensity and education coefficients for the group under 25 years, without impact on the conclusions, with there being no significant statistical relationships between the variables. A similar situation is found in the case of organizational skills for the over 50 age group. Autonomy becomes a statistically significant variable in the case of the 26–35 age group.

The analysis of the wellbeing of telework employees by age group is a novelty in the field, useful in managerial decisions. 

## 6. Conclusions

The complexity of the wellbeing concept involves specific assessments that include individual psychological, social, cognitive and physical aspects. As labor markets transition to more flexible working models based on digitization, particular influences may increase the problem. Complementarily, the contextualization of the pandemic COVID-19 conditions reveals characteristic perceptions. 

The main contribution of this paper consists in identifying and correlating several variables that define social and emotional aspects at an individual level (relational communication, emotional dimension, work intensity, organization, autonomy and work–life balance), proposing a model for determining their impact on wellbeing.

The results of the empirical study highlight that there is a statistically significant positive relationship between relational communication, emotional dimension, organization, work–life balance and wellbeing in the case of the telework regime. A statistically significant negative relationship is observed between work intensity and wellbeing. These results are in line with our expectations. The validity of the model is supported by partial results similar to those obtained by other researchers.

This research provides a useful tool for assessing wellbeing at work, considered an objective of human resource management, being a predictor of employee performance and quality of personal life. Focused analysis on sectors of activity and even on organizations allows decision makers to establish measures to correct certain issues and leverage them to enhance wellbeing.

The study allows the identification of both the exposures that harm employee wellbeing, as well as the positive aspects specific to the working conditions at the level of the entity, being useful to managers in taking measures in order to retain and motivate staff. By incorporating worker attitudes and behavior into the study of employee wellbeing, a useful tool is offered to HR specialists and managers for increasing the efficiency of human capital management. Furthermore, the results of the research are relevant for decision makers in establishing regulatory policies in a dynamic and complex macroeconomic environment. Policy objectives are mainly aimed at job creation and reducing unemployment and to a lesser extent, the quality of jobs (1). The use of telework offers people the opportunity to organize work around their lives, improving the quality of life. It is necessary to reconsider the inclusion of the quality of work in the concerns of policy-makers, as they ensure an improvement of wellbeing at the level of employees, both from a financial point of view and from the perspective of increasing self-confidence, diminishing social exclusion, improving social cohesion, with favorable impacts on the productivity and competitiveness of employers. Complementary to the effects on employees and employers, an improvement in wellbeing also leads to a decrease in pressure on the social system at the country level.

There are several limits that need to be addressed. Telework employees are a heterogeneous category. The paper includes exploratory research that allows us to explain, at the level of considered observations, the impact of social and emotional factors on the wellbeing of employees. The main limitation of this study derives from the subjectivity of the perception of the studied phenomenon and the bias of extending the conclusions to the entire population. Furthermore, considering the fact that the data collection was conducted online, the study investigated a convenience sample, susceptible to biases, raising the problem of generalization. In addition, this study is carried out in the particular context generated by the COVID-19 pandemic, the comparisons with studies conducted outside the inclusion criteria may emphasize different conclusions. The complex implications of this crisis and the approach of the social and emotional perspective determine specific results at the sample level. We did not test the results by performing comparative analyses on subsamples, for example, depending on the status (telework can be more attractive for parents, enables them to organize their job practices, work environment and time more efficiently, but less attractive for single people, who want a more intense socialization). Additionally, specific working populations are excluded; for example, the viewpoints of the employees in similar positions who work full-time in an employer’s location are not analyzed. This is a topic for the future studies.

The constructs and attributes such as attitude, personality and other similar variables raise the problem of the ordinality of their measurement and analysis. With reference to the methodology used, there are controversies regarding the option for ordinary least squares regression in the case of the analyses of responses to a Likert-type item. On the one hand, a common recommendation in the methodological literature is that the ordered categorical variables are analyzed using ordinal logistic regression [78,79,80]. The critical assumption of metrically scaled responses is thus avoided. On the other hand, several researchers have argued that the ordinary least squares regression is relevant if the variable uses a Likert scale. Thus, wellbeing, in its multiple dimensions: mental, emotional, financial, subjective, etc., was analyzed using ordinary least squares regression analysis [81,82,83,84,85,86,87]. Furthermore, a study analyzing a similar topic [88] looked at the importance of the methodology in the estimation of the determinants of happiness, concluding that there is little difference between running an ordinary least squares regression or taking an ordered logit or probit model. The wide-scale use of this practice can be motivated by the intention to substantiate the conclusions regarding the attitude by moving from “equivalent” hypotheses to data-based interpretations, with the limits arising from the particular nature of the variables.

Our interest in assessing the impact of changing working conditions and making the work schedule more flexible, as a condition of wellbeing growth, will be reflected in future studies that will address comparative wellbeing in the case of teleworking and carrying out the activity in employers’ premises. Given that policies to improve the quality of work life are included in the governance of welfare states, future studies will address comparisons across different welfare state regimes to identify their consequences on employee wellbeing. Additionally, the research was conducted during the COVID-19 pandemic when states supported employment through various job retention measures. The medical crisis, as well as the current geopolitical context, generate economic and financial crisis. Future research will aim to analyze the impact of telework after the period of major recession.

## Figures and Tables

**Table 1:** The endogenous and exogenous variables.

Variables	Type	Abbreviations	Items *
work intensity	exogenous	WI	6–9
relational communication	exogenous	RC	10–14
emotional dimension	exogenous	ED	15–18
organization	exogenous	ORG	19–21
autonomy	exogenous	AUT	22–25
work–life balance	exogenous	WLB	26–28
wellbeing	endogenous	WB	29

* question number in the questionnaire.

**Table 2:** Descriptive statistics of variables.

Variable	WI	RC	ED	ORG	AUT	WLB	WB
Total							
Mean	3.24	4.15	3.87	4.30	3.47	3.68	3.82
Median	3.25	4.20	4.00	4.33	3.50	3.67	4.00
Maximum	5.00	5.00	5.00	5.00	4.75	5.00	5.00
Minimum	1.00	1.20	1.75	2.33	1.50	1.00	1.00
Std. Dev.	1.05	0.72	0.78	0.60	0.64	0.80	0.88
Female							
Mean	3.22	4.10	3.84	4.23	3.42	3.59	3.78
Median	3.25	4.20	4.00	4.33	3.50	3.67	4.00
Maximum	5.00	5.00	5.00	5.00	4.75	5.00	5.00
Minimum	1.00	1.20	1.75	2.33	1.75	1.00	1.00
Std. Dev.	1.04	0.73	0.80	0.62	0.63	0.82	0.86
Male							
Mean	3.29	4.26	3.93	4.43	3.58	3.86	3.89
Median	3.25	4.40	4.00	4.33	3.75	4.00	4.00
Maximum	5.00	5.00	5.00	5.00	4.50	5.00	5.00
Minimum	1.00	1.20	2.00	3.00	1.50	1.67	1.00
Std. Dev.	1.09	0.68	0.73	0.51	0.64	0.72	0.90

Source: Elaborated by the authors in the EViews program.

**Table 3:** Correlations between variables.

	WI	RC	ED	ORG	AUT	WLB	WB
WI	1						
RC	0.116 *	1					
ED	0.599 ***	0.317 ***	1				
ORG	0.149 **	0.432 ***	0.335 ***	1			
AUT	0.008	0.448 ***	0.137 **	0.432 ***	1		
WLB	0.446 ***	0.322 ***	0.549 ***	0.339 ***	0.280 ***	1	
WB	0.015	0.393 ***	0.237 ***	0.506 ***	0.334 ***	0.295 ***	1

Note: * *p* < 0.05; ** *p* < 0.01; *** *p* < 0.001. Source: Elaborated by the authors in the EViews program.

**Table 4:** Results of the regression—model 1.

Dependent Variable: WB				
	OLS		OLM	
Variable	Coefficient	Prob.	Coefficient	Prob.
WI	−0.163	0.001	−0.438	0.0001
RC	0.229	0.001	0.678	0.0001
ED	0.147	0.022	0.324	0.0617
ORG	0.441	0.001	1.167	0.0000
AUT	0.097	0.139	0.306	0.0865
WLB	0.151	0.006	0.418	0.0051

Note: hypothesis testing—homoskedasticity: White test, Prob. Chi-square 0.13; non-correlation of errors: Lagrange Multiplier test, Prob. Chi-square 0.75; non-collinearity of variables, VIF values of independent variables less than 4: WI 1.63, RC 1.49, ED 2.05, ORG 1.43, AUT 1.47 and WLB 1.63. Source: Elaborated by the authors in the EViews program.

**Table 5:** Results of the regression—model 2.

Dependent Variable: WB				
Variable	Coefficient			
	OLS		OLM	
	Female	Male	Female	Male
WI	−0.1269 *	−0.1605 *	−0.3449 *	−0.4361 *
RC	0.1246 *	0.5961 ***	0.3735 *	1.7144 ***
ED	0.1987 **	−0.041	0.503 *	−0.2757
ORG	0.4229 ***	0.3439 *	1.1275 ***	1.0405 **
AUT	0.1224	−0.056	0.3612	−0.1422
WLB	0.1981 **	0.0101	0.5378 **	0.1563

Note: * *p* < 0.05; ** *p* < 0.01; *** *p* < 0.001. Note: hypothesis testing—homoskedasticity: Breusch–Pagan–Godfrey test, Prob. Chi-square 0.16 for men and 0.42 for women; non-correlation of errors: Lagrange Multiplier test, Prob. Chi-square 0.16 for men and 0.17 for women; non-collinearity of variables, VIF values of independent variables less than 10: WI 1.68 men/1.67 women, RC 2.12 men/1.36 women, ED 1.86 men/2.19 women, ORG 1.35 men/1.48 women, AUT 1.63 men/1.43 women and WLB 1.40 men/1.76 women. Source: Elaborated by the authors in the EViews program.

**Table 6:** Results of the regression—model 3 OLS.

Variable	Coefficient			
	Age up to 25	Age between 26–35	Age between 36–50	Age over 50
WI	−0.0437	−0.1026	−0.1186 ^#^	−0.0959
RC	0.4548	0.126	0.2844 **	0.4424 ***
ED	0.0597	−0.0939	0.1149	0.1347
ORG	0.0939	0.6658 ***	0.3923 ***	−0.0223
AUT	0.2707	−0.2149	0.1669 ^#^	0.2796 *
WLB	−0.0671	0.1245	0.0705	0.482 ***

Note: * *p* < 0.05; ** *p* < 0.01; *** *p* < 0.001, ^#^ *p* < 0.1 Source: Elaborated by the authors in the EViews program.

**Table 7:** Results of the regression—model 3 OLM.

Variable	Coefficient			
	Age up to 25	Age between 26–35	Age between 36–50	Age over 50
WI	0.0139	−0.2724	−0.3792 *	−0.3259
RC	0.9334	0.4939	0.7472 **	1.6290 ***
ED	−0.1317	−0.4057	0.3341	0.3922
ORG	0.5483	1.8498 ***	1.1065 ***	0.1072
AUT	0.8449	−0.7330 *	0.4874 *	1.0135 *
WLB	−0.1535	0.4148	0.1972	1.7391 ***

Note: * *p* < 0.05; ** *p* < 0.01; *** *p* < 0.001. Source: Elaborated by the authors in the EViews program.

## Data Availability

Not applicable.
